# D-β-aspartyl residue exhibiting uncommon high resistance to spontaneous peptide bond cleavage

**DOI:** 10.1038/srep21594

**Published:** 2016-02-15

**Authors:** Kenzo Aki, Emiko Okamura

**Affiliations:** 1 Faculty of Pharmaceutical Sciences, Himeji Dokkyo University, 7-2-1 Kamiohno, Himeji 670-8524, Japan

## Abstract

Although L-amino acids were selected as main constituents of peptides and proteins during chemical evolution, D-aspartyl (Asp) residue is found in a variety of living tissues. In particular, D-β-Asp is thought to be stable than any other Asp isomers, and this could be a reason for gradual accumulation in abnormal proteins and peptides to modify their structures and functions. It is predicted that D-β-Asp shows high resistance to biomolecular reactions. For instance, less reactivity of D-β-Asp is expected to bond cleavage, although such information has not been provided yet. In this work, the spontaneous peptide bond cleavage was compared between Asp isomers, by applying real-time solution-state NMR to eye lens αΑ-crystallin 51–60 fragment, S^51^LFRTVLD^58^SG^60^ and αΒ-crystallin 61–67 analog, F^61^D^62^TGLSG^67^ consisting of L-α- and D-β-Asp 58 and 62, respectively. Kinetic analysis showed how tough the uncommon D-β-Asp residue was against the peptide bond cleavage as compared to natural L-α-Asp. Differences in p*K*_a_ and conformation between L-α- and D-β-Asp side chains were plausible factors to determine reactivity of Asp isomers. The present study, for the first time, provides a rationale to explain less reactivity of D-β-Asp to allow abnormal accumulation.

It is well known that L-amino acids were selected as main constituents of peptides and proteins during chemical evolution. However, D-amino acid residues have been found in a variety of living tissues^1^,^2^,^3^. Most typical is a D-aspartyl (Asp) residue that has been detected in aged lens^1^,^4^,^5^,^6^, tooth^7^,^8^, aorta^9^, brain^10^, bone^3^,^11^, and ligament^12^. D-Asp is produced by racemization of natural L-Asp residue, the racemization accompanied by isomerization from natural α-Asp to uncommon β-Asp via a succinimide intermediate^13^. If we consider these pathways, D-β-Asp residue is thought to be stable than any other Asp isomers (L-α-, L-β-, and D-α-Asp). This could be a most plausible reason for the gradual accumulation of D-β-Asp residue in a variety of proteins and peptides^14^,^15^. Aged eye lens αΑ- and αΒ-crystallins are typical examples, in which Asp 58 and 151 in αΑ-crystallin as well as Asp 36 and 62 in αΒ-crystallin are converted from L-α- to D-β-Asp with high probability^4^,^5^,^14^.

It is predicted that racemization and isomerization of natural L-α-amino acids could modify the structure and function of various proteins. In fact, most of enzymes and receptors can recognize the difference in molecular chirality in a living body. There is also a possibility that biomolecular reactions are modified by the presence of unusual amino acid isomers. For instance, peptide bonds are spontaneously cut off at sites next to normal L-α-Asp and asparagine residues^16^,^17^,^18^. The bond cleavage reaction is, in particular, effective in avoiding formation and accumulation of undesired peptide bonds including uncommon amino acid residues. To the contrary, if peptide bonds get high resistance to the cleavage reaction, unusual amino acid residues gradually accumulate in a living body to allow abnormal stage of protein structures and functions. Considering that D-β form of Asp residue is most stable among Asp isomers, one can imagine that D-β-Asp shows an ability to resist spontaneous peptide bond cleavage. To our best of knowledge, however, no information has been provided about the peptide bond cleavage of unusual D-β amino acid residues.

In this work, the kinetics of peptide bond cleavage at L-α- and D-β-Asp residues was compared for the first time, to explore how tough the uncommon D-β form of Asp residue is against the peptide bond cleavage as compared to natural L-α-Asp. Solution-state ^1^H NMR was applied for this purpose, to observe the cleavage reaction in real time. A human lens αΑ-crystallin 51–60 fragment, S^51^LFRTVLD^58^SG^60^ (αΑ51–60) and αΒ-crystallin 61–67 analog, F^61^D^62^TGLSG^67^ (αΒ61–67) composed of L-α- and D-β-Asp (Fig. 1) were synthesized as model peptides. The αΑ51–60 and αΒ61–67 sequences were selected because Asp58 in αΑ- and Asp62 in αΒ-crystallin have been found to be highly converted from L- to D-isomer in aged human lenses^4^,^14^. Considering that 1–58 region of αΑ-crystallin as well as 1–65 of αΒ-crystallin display inherent conformational flexibility^19^, the choice of the present short sequences, αΑ51–60 and αΒ61–67, is rational enough to represent the corresponding sequences in natural α-crystallins.

When the peptide bond is cut off between Asp58 and Ser59 in 51–60 αΑ-crystallin fragment, non-terminal Asp58 residue is converted to C-terminal Asp58. The product of C-terminal L-Asp58 or D-Asp58 is expected from non-terminal L-α- or D-β-Asp58. In addition, an equimolecular amount of N-terminal Ser59 is produced as a result of the bond cleavage next to L-α- and D-β-Asp58. Therefore, we can simultaneously analyze the bond cleavage reaction from (i) the decrease in non-terminal L-α- or D-β-Asp58 as a reactant, (ii) the increase in C-terminal L- or D-Asp58 as a product, and (iii) the increase in N-terminal Ser59 of Ser59-Gly60 as another product. Similarly, the bond cleavage in αΒ61–67 sequence can also be monitored by (i) the decrease in non-terminal L-α-/D-β-Asp62 as well as non-terminal Thr63 as a reactant, (ii) the increase in C-terminal L- or D-Asp62 as a product, and (iii) the increase in N-terminal Thr63 of 63–67 fragment as another product. Utilizing the ability of high-resolution solution-state NMR to distinguish (i), (ii), and (iii) in real time, here we evaluate how fast the peptide bond cleavage proceeds in the presence of L-α- and D-β-Asp residues. The comparison demonstrates that both D-β-Asp isomers in αΑ51–60 and αΒ61–67 show high resistance to peptide bond cleavage, that may cause undesired accumulation of D-β-Asp residue in peptides and proteins with time.

## Results

### Difference in ^1^H NMR signals between peptides of Asp isomers

Let us compare the ^1^H NMR spectra of αA-crystallin 51–60 fragment (αA51–60) containing L-α- or D-β-Asp58 isomers. In this work, each fragment was dissolved in acetate buffer/D_2_O (pD 4.0) and subject to NMR measurement at 70 °C. The conditions pD 4.0 and 70 °C were adopted to avoid undesired side reactions such as racemization and isomerization of Asp residue via a succinimide intermediate.

**Figure 2:**
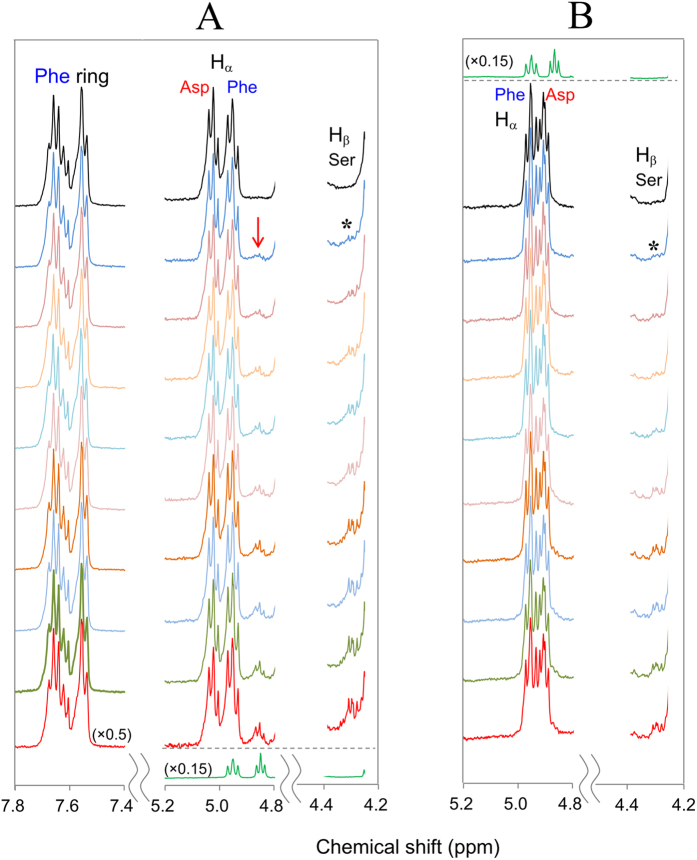
Real time ^1^H-NMR spectra of αA-crystallin 51–60 fragment containing L-α- and D-β-Asp58. Panel (**A**) illustrates ^1^H-NMR spectra for the peptide containing L-α-Asp58 immediately after and 5, 7, 10, 11, 12, 14, 16, 17 and 20 h after heating at 70 °C (from top to bottom). Panel (**B**) shows ^1^H-NMR spectra for 51–60 peptide containing D-β-Asp58 immediately after and 5, 6, 7, 8, 17, 18, 19, 20 and 21 h after heating at 70 °C. The ^1^H-NMR spectra of 51–58 peptide containing C-terminal L-and D-Asp58 are shown in green at the bottom of panel (**A**) and the top of panel (**B**), respectively. Each panel shows the region of Phe53 ring protons (7.8–7.4 ppm), H_α_ of Phe53 and Asp58 (5.2–4.8 ppm), and H_β_ of Ser59 (4.4–4.2 ppm).

**Figure 3:**
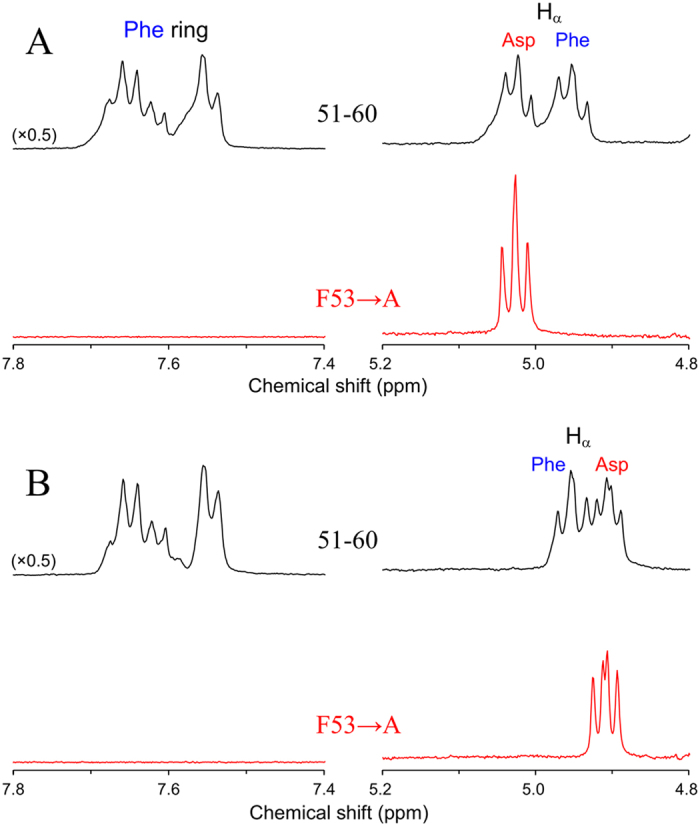
Comparison of the ^1^H NMR spectra of (**A**) L-α- and (**B**) D-β-Asp58 isomers of αA-crystallin 51–60 fragment (in black) and its F53→A derivative (in red) at 70 °C. Each peptide was dissolved in 50 mM acetate buffer/D_2_O (pD 4.0).

It was found that ^1^H NMR spectra showed a difference between the peptides containing L-α- and D-β-Asp isomers. As indicated by the top spectrum (in black) in Fig. 2A, the H_α_ proton signal of non-terminal L-α-Asp58 was observed at 5.1–5.0 ppm. The assignment was confirmed by the absence of the signal in a 51–58 fragment peptide, S^51^LFRTVLD^58^ containing C-terminal L-Asp58; see the bottommost spectrum (in green). For non-terminal D-β-Asp58, on the other hand, the H_α_ signal moved to 4.9–4.85 ppm; see the spectrum (in black) in Fig. 2B. The assignment was also confirmed by the absence of the signal in a 51–58 fragment composed of C-terminal D-Asp58 (in green) as illustrated at the top of Fig. 2B. It is noted that the Hα peak of D-β-Asp58 is observed at higher magnetic field as compared to Phe53 H_α_ at 5.0–4.9 ppm; notice that the assignment of Phe53 H_α_ is confirmed by comparing ^1^H NMR spectra of αA51–60 and its derivative where Phe53 is substituted to Ala53 (Fig. 3). The location of D-β-Asp58 Hα at high magnetic field is in contrast to the case of L-α-Asp58 (panel A) in which the Hα signal is observed at lower magnetic field than Phe53 H_α_.

### Real-time NMR of peptide bond cleavage at L-α- and D-β-Asp

Figure 2A,B illustrate how the ^1^H NMR spectra of αA51–60 containing L-α- and D-β-Asp58 were varied at 70 °C in real time. For the fragment of L-α-Asp58, a new peak appeared at ~4.85 ppm after 2 h. Afterwards, the peak increased in intensity with time, as indicated by the red arrow in panel A. Simultaneously, the H_α_ signal of non-terminal L-α-Asp58 at 5.1–5.0 ppm was gradually lost. Such behavior of Asp58 was in contrast to the spectral change of Phe53 residue, where signals of ring proton (7.67–7.54 ppm) and H_α_ (around 4.95 ppm) were kept constant (see the signal intensity change of Phe53 ring proton in the supplement Figure S1). The new peak was assigned to C-terminal L-Asp58, as compared to the bottommost spectrum of 51–58 fragment, S^51^LFRTVLD^58^ (in green). Totally, the observed spectral changes show that the non-terminal L-α-Asp58 of αA51–60 was gradually converted to C-terminal L-Asp58, as a result of spontaneous peptide bond cleavage next to L-α-Asp58. It is emphasized that Asp58 residue is only the site for bond cleavage reaction. This was confirmed by HPLC elution profile that showed a product peak with masses ([M+H]^+^) of 950.5, the value consistent with the theoretical one of the 51–58 fragment, 950.53.

When the peptide bond is cut off between Asp58 and Ser59, a dipeptide, Ser59-Gly60 is also produced. The production of Ser59-Gly60 leads to an increase in N-terminal Ser59 with time. In fact, a signal was observed at ~4.31 ppm (indicated by *in panel A) and increased with time. The signal is assignable to H_β_ of N-terminal L-Ser59 because such peak was absent for the spectrum of 51–58 fragment (in green). Thus we can capture all the spectral changes associated with the peptide bond cleavage next to L-α-Asp58; (i) the decrease in non-terminal L-α-Asp58, (ii) the increase in C-terminal L-Asp58, and (iii) the increase in N-terminal L-Ser59 in panel A.

To examine whether peptide bond cleavage is modified by the presence of D-β-Asp58, the ^1^H NMR spectra of αA51–60 containing D-β-Asp58 were observed at pD 4.0 and 70 °C. In analogy with L-α-Asp peptide, the bond cleavage was analyzed in terms of (i) the loss of non-terminal D-β-Asp58, (ii) the increase in C-terminal D-Asp58, and (iii) the increase in N-terminal L-Ser59. As illustrated in Fig. 2B, the intensities of the H_α_ signal of non-terminal D-β-Asp58 (around 4.91 ppm) were slightly decreased as compared to the constant Phe53 H_α_ (around 4.95 ppm). However, no significant increase of the C-terminal D-Asp58 signal was found at around 4.87 ppm. This was evident from the comparison to the spectrum of 51–58 fragment peptide, S^51^LFRTVLD^58^ containing C-terminal D-Asp58, shown at the top (in green) of panel B. The result means that the peptide bond cleavage was limited by the presence of uncommon D-β-Asp58. If this is the case, the increase in N-terminal L-Ser59 should also be suppressed. Actually, the H_β_ signal intensity of N-terminal Ser59 at 4.3 ppm (*in panel B) was less increased with time, as compared to the case of L-α-Asp58 isomers (panel A). All results show that the presence of uncommon D-β-Asp58 interferes with the spontaneous peptide bond cleavage, which may accumulate undesired D-β-Asp58 residue in the peptide.

### Kinetics of peptide bond cleavage at L-α- and D-β-Asp

In the previous section, we have qualitatively demonstrated that uncommon D-β-Asp58 in αA51–60 shows high resistance to peptide bond cleavage next to Asp. To quantify how D-β-Asp residue interferes with the bond cleavage reaction, we determined the rate constants of the bond cleavage at L-α- and D-β-Asp58 by using real-time signal intensity changes of reactants and products. The kinetic analysis is valid because NMR signal intensity is proportional to the concentration of each reactant and product in the present cleavage reaction.

**Figure 4:**
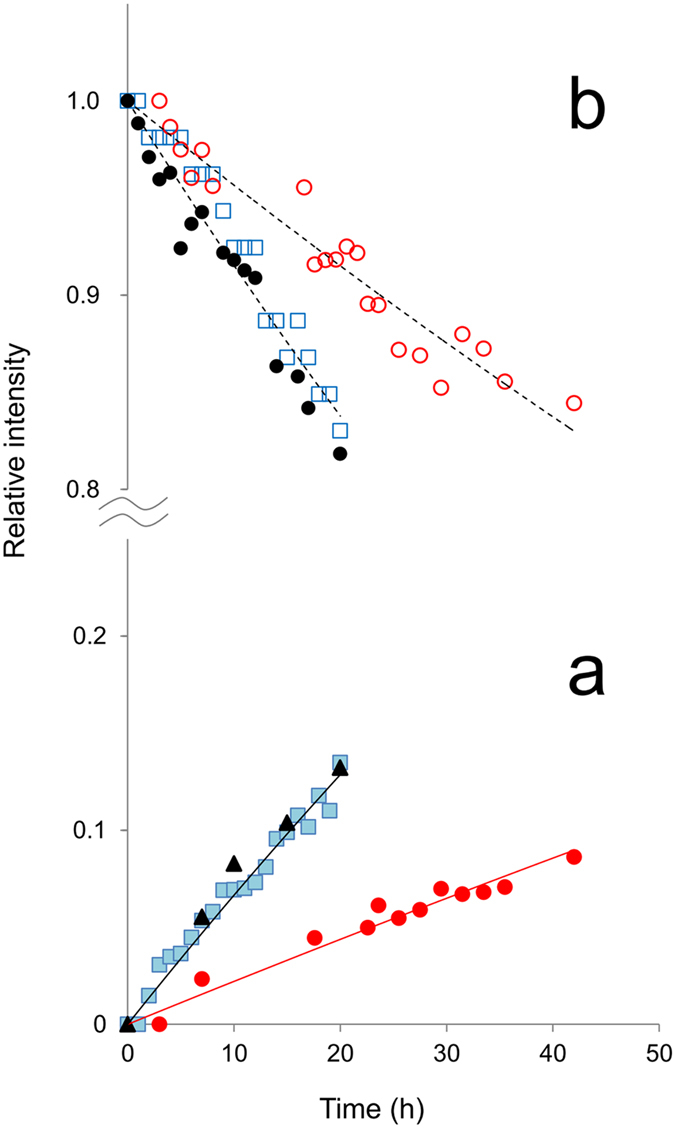
Real time NMR signal intensity change accompanied by the peptide bond cleavage at L-α- and D-β-Asp58 of αA-crystallin 51–60 fragment. The increase in the products C-terminal L-Asp58 (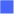) of 51–58 fragment and N-terminal Ser59 of Ser59-Gly60 (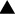) from the reactant L-α-Asp 51–60 is plotted in (**a**), together with the increase in N-terminal Ser59 (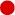) from the reactant D-β-Asp 51–60. Each symbol designates integral intensity of the respective signal. In (**b**), the decrease of the reactant non-terminal L-α-Asp58 evaluated from integral intensities (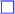) and peak heights (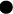) is shown, as well as that of non-terminal D-β-Asp58 estimated from peak heights (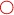). All values are relative to the initial intensities. Black and red solid lines in (**a**) are the resulting curves obtained by fitting equation 2 to the respective experimental values, from which the rate constants *k* were estimated. Dashed lines in (**b**) are only guide for eyes.

First, the increase in the cleavage products of L-α-Asp peptide was quantified by using the integral intensities of H_α_ signal of C-terminal L-Asp58 at 4.85 ppm as well as the intensities of N-terminal Ser59 H_β_ at 4.3 ppm, as described in the *Methods* section. The result was plotted as filled squares (L-Asp58) and triangles (Ser59) in Fig. 4a, relative to the initial concentration of the reactant. On the other hand, the loss of the reactant was evaluated by using H_α_ signal of non-terminal L-α-Asp58 at 5.1–5.0 ppm. Because H_α_ signal of non-terminal L-α-Asp58 was partly overlapping with Hα of Phe53 at around 4.95 ppm, the loss of non-terminal L-α-Asp58 was quantified by subtracting constant (time-independent) intensity of Phe53 H_α_ from the sum of the H_α_ signal intensities of non-terminal L-α-Asp58 and Phe53, and plotted as open squares in Fig. 4b.

Next, to quantify how D-β-Asp modifies the peptide bond cleavage, the signal intensities of αA51–60 consisting of D-β-Asp58 were also analyzed as a function of time. Because H_α_ signal of the non-terminal D-β-Asp58 was severely overlapping with Phe53 (Fig. 2B), the relative intensity of the non-terminal D-β-Asp58 was estimated from the peak height at 4.91 ppm (open circles in Fig. 4b). This estimation was justified, since relative intensities from the peak height of non-terminal L-α-Asp58 H_α_ at 5.02 ppm (filled black circles) were found to be consistent with those from integral intensities (open squares), as shown in Fig. 4b. Further, it was not possible to estimate the signal intensity of C-terminal D-Asp58 at 4.87 ppm because the signal was weak and overlapped with the non-terminal D-β-Asp58. Therefore, the amount of C-terminal D-Asp58 was replaced by the concentration of N-terminal Ser59 (filled red circles in Fig. 4a), the equivalent product of C-terminal D-Asp58 from the bond cleavage reaction.

The comparison of time-dependent intensity changes between L-α and D-β peptides demonstrates that the reactivity of D-β-Asp residue to peptide bond cleavage is different from that of L-α-Asp. As shown in Fig. 4b, the loss of non-terminal D-β-Asp58 is slower than that of L-α-Asp58. This means that D-β-Asp shows high resistance to peptide bond cleavage, as qualitatively stated in the previous section. Less reactivity of uncommon D-β-Asp58 residue is also confirmed by rather small increase of the cleavage product N-terminal Ser59 as compared to the products, L-Asp and Ser59 from L-α-Asp58 isomer (Fig. 4a).

Now let us focus on the rate constants for spontaneous peptide bond cleavage at L-α- and D-β-Asp58. When the cleavage reaction is treated as first-ordered, the rate of the product increase follows the rate equation as:


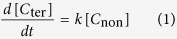
where [*C*_ter_] is the concentration of a product, C-terminal Asp58 or N-terminal Ser59, and [*C*_non_] is the concentration of a reactant, non-terminal Asp58, respectively. *k* is the rate constant for the bond cleavage reaction at L-α- and D-β-Asp58. According to equation 1, the concentrations of terminal Asp58 and Ser59, [*C*_ter_] are given by


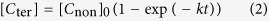
where [*C*_non_]_0_ is the initial concentrations of non-terminal L-α- and D-β-Asp58. Thus the rate constant *k* can be determined by fitting equation 2 to the respective experimental values of C-terminal Asp58 and N-terminal Ser59 obtained.

In Fig. 4a, the fitting results were illustrated as black and red solid curves for the cleavage products from non-terminal L-α-Asp58 and D-β-Asp58 fragments, respectively. The rate constants *k* were estimated to be (6.9 ± 0.5) × 10^−3 ^h^−1^ for L-α-Asp and (2.2 ± 0.4) × 10^−3 ^h^−1^ for D-β-Asp residue. The reactivity of uncommon D-β-Asp was less than one-third of natural L-α-Asp in an αA-crystallin fragment.

### Bond cleavage reaction in αB-crystallin 61–67 sequence

 In the previous section, we showed that D-β-Asp58 residue was less reactive to the peptide bond cleavage as compared to natural L-α-Asp58 in an αA-crystallin fragment. Such less reactivity of D-β-Asp keeps D-β-peptide stable and that is a plausible reason for gradual accumulation of D-β-Asp with age. To confirm the hypothesis, we have compared the cleavage kinetics of an αΒ-crystallin 61–67 derivative, F^61^D^62^TGLSG^67^ (αB61–67), composed of L-α- and D-β-Asp62 as a second model sequence.

**Figure 5:**
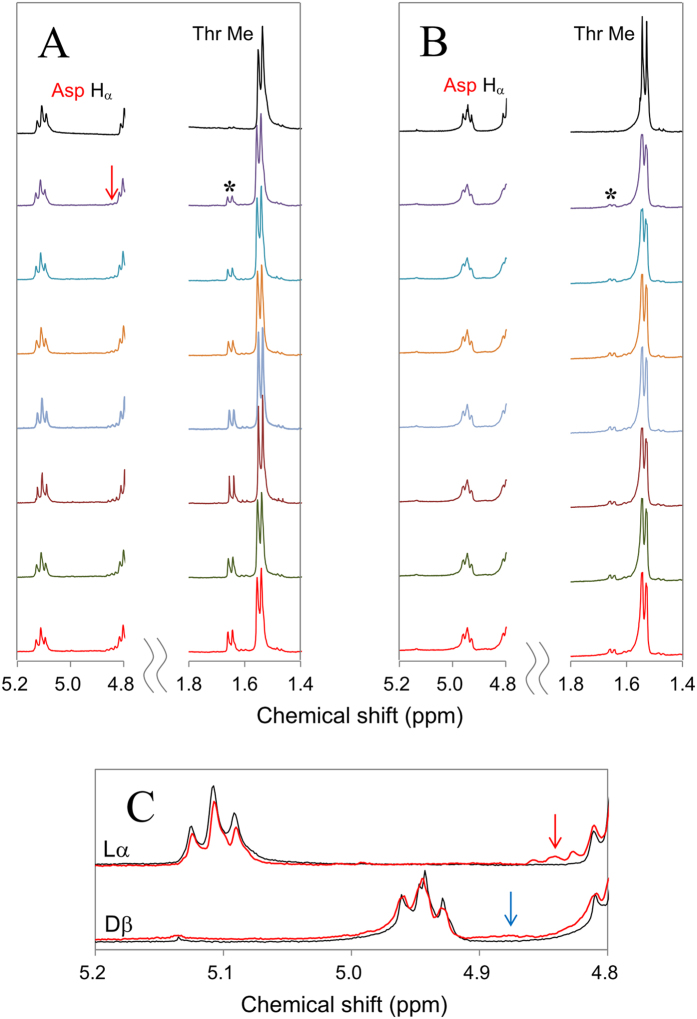
Real time ^1^H-NMR spectra of αB-crystallin 61–67 analog containing L-α- and D-β-Asp62. Panel (**A**) illustrates ^1^H-NMR spectra for the peptide containing L-α-Asp62 immediately after and 16, 20, 24, 28, 32, 35, and 38 h after heating at 70 °C (from top to bottom). Panel (**B**) shows ^1^H-NMR spectra for 61–67 peptide containing D-β-Asp62 immediately after and 23, 26, 30, 32, 34, 36, and 38 h after heating at 70 °C. Each panel shows Asp62 H_α_ (5.2–4.8 ppm) and Thr63 methyl regions (1.8–1.4 ppm). In panel (**C**), the H_α_ region of L-α- and D-β-Asp62 immediately after (in black) and 38 h after heating (in red) is expanded in upper and lower traces, respectively.

In Fig. 5A,B, the real-time ^1^H NMR spectra of αB61–67 composed of L-α- and D-β-Asp62 were compared. The H_α_ region of L-α- and D-β-Asp62 was also expanded in Fig. 5C. When the peptide bond is cleaved between Asp62 and Thr63, the non-terminal Asp62 is converted to C-terminal Asp62, together with the production of an equimolecular amount of N-terminal Thr63. Thus the bond cleavage reaction can be analyzed by (i) the decrease in non-terminal L-α- or D-β-Asp62 as well as non-terminal Thr63 as a reactant, (ii) the increase in C-terminal L- or D-Asp62 as a product, and (iii) the increase in N-terminal Thr63 of 63–67 fragment as another product. In fact, as illustrated by upper traces in Fig. 5C, the H_α_ signal of non-terminal L-α-Asp62 around 5.1 ppm was decreased and converted to C-terminal L-Asp62 around 4.85 ppm (red arrow). At the same time, the doublet assigned to N-terminal Thr63 methyl at 1.66 and 1.64 ppm, indicated by *in Fig. 5A, was gradually increased from non-terminal Thr63 at 1.56 and 1.54 ppm. All results show that the bond cleavage also proceeds at L-α-Asp62 in αB61–67, similar to the case in L-α-Asp58 in αA51–60.

For D-β-Asp62 in αB61–67 sequence, the peptide bond cleavage was found to be slower than L-α-Asp62. As shown in Fig. 5B, the amount of N-terminal Thr63 (*) as a cleavage product was not so much, as compared to L-α-Asp62 isomers in Fig. 5A. Further, the production of C-terminal D-Asp62 around 4.87 ppm, indicated by blue arrow in Fig. 5C, was not marked even 38 h after heating; see lower traces in Fig. 5C. This means that D-β-Asp62 in αB61–67 also shows high resistance to peptide bond cleavage, quite similar to D-β-Asp58 in αA51–60 sequence.

**Figure 6:**
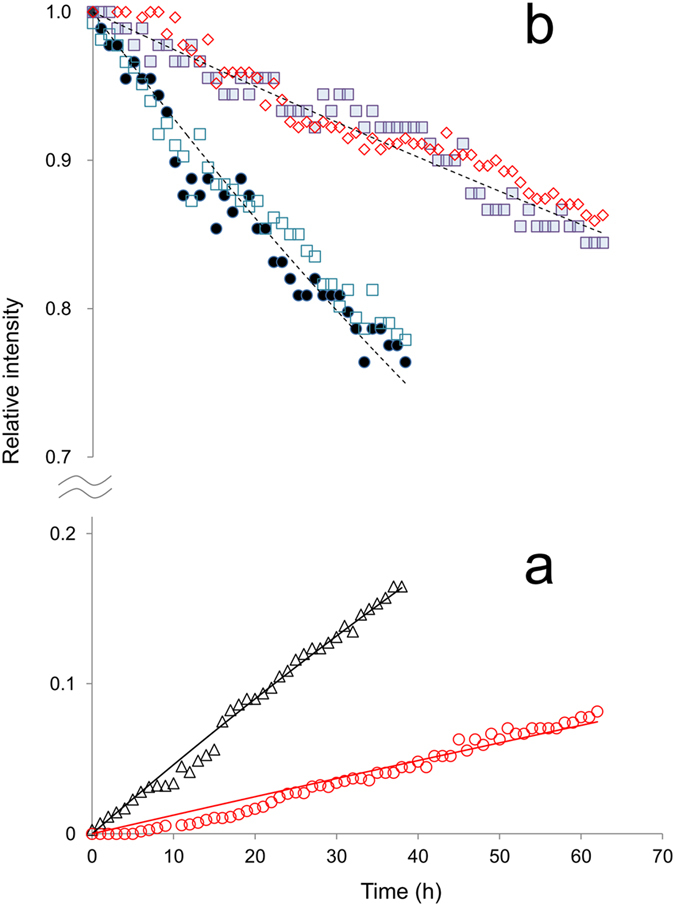
Real time NMR signal intensity change accompanied by the peptide bond cleavage at L-α- and D-β-Asp62 of αB-crystallin 61–67 analog. The increase in the product N-terminal Thr63 (Δ) of 63–67 fragment from the reactant L-α-Asp 61–67 is plotted in (**a**), together with the increase in N-terminal Thr63 (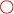) from the reactant D-β-Asp 61–67. Each symbol designates the integral intensity of the respective signal. In (**b**), the decrease of the reactant evaluated from the integral intensities of non-terminal L-α-Asp62 (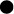) and Thr63 (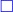) in L-α-Asp 61–67 is shown, as well as that of non-terminal D-β-Asp62 (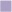) and Thr63 (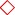) in D-β-Asp 61–67. All values are relative to the initial intensities. Black and red solid lines in (**a**) are the resulting curves obtained by fitting equation 2 to the respective experimental values, from which the rate constants *k* were estimated. Dashed lines in (**b**) are only guide for eyes.

The time-dependent concentration changes in Fig. 6a,b quantitatively validate less reactivity of D-β-Asp62 in αB61–67. As shown in Fig. 6a, the amount of the product Thr63 from D-β-Asp62 cleavage (red circles) is less than the one from L-α-Asp62 (black triangles). The decrease of non-terminal D-β-Asp62 (filled squares) and Thr63 (diamonds) as a reactant is also suppressed, as compared to non-terminal L-α-Asp62 (filled circles) and Thr63 (open squares) in L-α-Asp62 isomers. Such less reactivity of D-β-Asp62 is evident from the rate constant *k* of (1.3 ± 0.2) × 10^−3 ^h^−1^ as compared to (4.7 ± 0.9) × 10^−3 ^h^−1^ for L-α-Asp62, that is obtained by fitting equation 2 to the respective experimental values.

## Discussion

**Figure 7:**
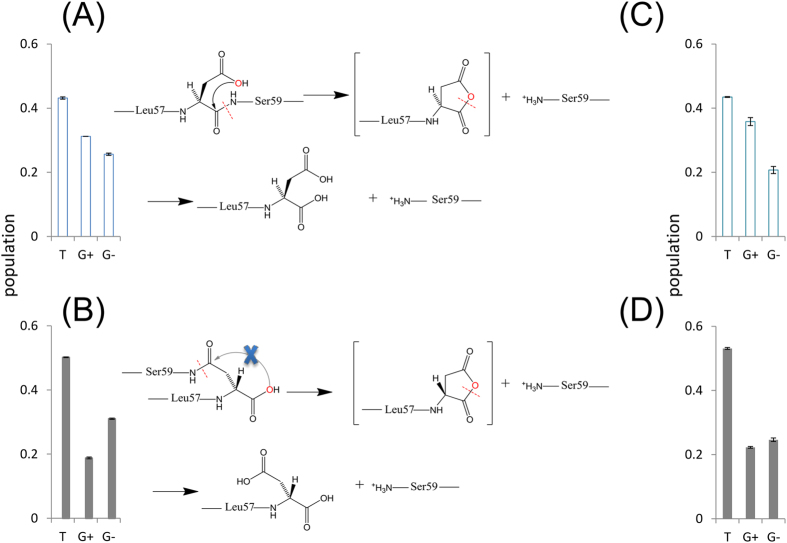
Population of the side chain conformers of (**A**) L-α- and (**B**) D-β-Asp58 in αA51–60, and (**C**) L-α- and (**D**) D-β-Asp62 in αB61–67 peptides. In (**A,B**), the reaction pathways of peptide bond cleavage at L-α- and D-β-Asp58 in αA51–60 are also shown, based on the respective Asp side chain conformers. Notice that the trans conformer of D-β-Asp side chain (**B**) interferes with the formation of cyclic anhydride intermediate (see text).

As described in the previous section, we demonstrated that uncommon D-β-Asp was less active to peptide bond cleavage (degradation) than L-α-Asp residue. Here we consider why such difference in reactivity was found in L-α- and D-β-Asp residues. Judging from that peptide bond cleavage proceeds via a cyclic anhydride intermediate^17^ as shown in the reaction scheme of Fig. 7, the following two factors are taken into consideration. One is that p*K*_a_ of L-α- and D-β-Asp side chain carboxyl is different from each other. In fact, it has been previously reported that p*K*_a_ of β-Asp side chain carboxyl group is 3.3 at 60 °C, lower than the value of α-Asp, 4.0^20^. At pD 4 in this study, therefore, the carboxyl group of D-β-Asp side chain is more ionized than L-α-Asp. When the bond cleavage undergoes from unionized state of carboxyl group as illustrated in Fig. 7, the ionized carboxylate in D-β-Asp side chain is disadvantageous for the cleavage reaction. The effectiveness of unionized carboxylic side chain in Asp for the bond cleavage reaction was also confirmed by our preliminary data obtained under different pH conditions. Thus, the p*K*_a_ of Asp carboxylic side chain is thought to explain the difference in the cleavage rate between Asp isomers.

The second reason is the difference in staggered side chain conformers between L-α- and D-β-Asp isomers. We have previously studied the population of Asp side chain conformation in αA-crystallin mimic 55–65 peptide composed of Asp58 isomers by high-resolution solution NMR^21^. It has been found that D-β-Asp58 shows the highest population of trans (T) conformer. The dominance of T conformer in D-β-Asp58 was also the case in the present αA51–60, S^51^LFRTVLD^58^SG^60^, as shown in the bar charts in Fig. 7A,B. In 51–60 fragment, the cleavage reaction proceeds as : (i) when the carbonyl carbon (C_CO_) of Asp58 peptide bond is nucleophilically attacked by hydroxyl oxygen of carboxylic side chain (O_COOH_), a cyclic anhydride intermediate is formed; (ii) another fragment Ser59-Gly60 is also cleaved as a result of intramolecular cyclization; and (iii) the 51–58 fragment including C-terminal Asp58 is finally produced by hydrolyzing cyclic anhydride intermediate; see the reaction scheme in Fig. 7. The trans conformer of D-β-Asp58 side chain is thought to be disadvantageous for such reaction pathway because the long distance between C_CO_ and O_COOH_ interferes with the nucleophilic attack of O_COOH_ on C_CO_ to initiate intramolecular cyclization (Fig. 7B). This is contrasting to gauche conformers of L-α-Asp58 side chain (Fig. 7A) where the distance between O_COOH_ and C_CO_ is short enough to allow intramolecular cyclization. The stabilization against the bond cleavage may occur via “autosolvation” reported by Pályi *et al*.^22^,^23^.

The situation was quite similar in αB-crystallin 61–67 sequence. As shown in the bar charts in Fig. 7C,D, D-β-Asp62 shows the highest population of T conformer to interfere with the intramolecular cyclization to initiate bond cleavage reaction. It has also been proposed that D-β-Asp shows low activity to racemization and isomerization^24^. The present study provides a rationale to explain less reactivity of D-β-Asp to allow abnormal accumulation with time.

## Methods

### Peptide synthesis

Isomers of αA51–60 and αB61–67 composed of L-α- and D-β-Asp residues were synthesized by Fmoc solid-phase chemistry using an automated solid-phase peptide synthesizer (Shimadzu PSSM-8). Fmoc-amino acids from Watanabe Chemical Industries (Hiroshima, Japan) were used. Crude peptides were purified by RP-HPLC using a C18 column (Capcellpak C18 ACR, 10 × 250 mm; Shiseido) with a linear gradient of 10–60% (for αA51–60) and 5–55% acetonitrile (for αB61–67) for 60 min in the presence of 0.1% TFA at a flow rate of 3.0 mL/min with monitoring at 215 nm. HPLC grade solvent was used to confirm the purity of the peptide. The purity of each peptide was confirmed to be >98% by RP-HPLC and MALDI-TOF MS or ESI-MS. The masses ([M+H]^+^) observed for the protonated precursor ions of αA51–60 and αB61–67 were 1094.6 and 696.1. These values were consistent with the theoretical ones, 1094.58 and 696.32, respectively.

### Real-time NMR measurement

Real-time ^1^H-NMR measurements were carried out on 400 MHz spectrometer (JEOL ECA400) equipped with a super-conducting magnet of 9.4 T. A high sensitivity probe (JEOL, NM40T10A/AT) for 10-mm diameter tube was used to obtain good S/N ratio. About 3 mg of αA51–60 and αB61–67 consisting of L-α- or D-β-Asp residue was dissolved in 4 ml of 50 mM acetate buffer/D_2_O (pD 4.0) and subject to NMR measurement at 70 °C. D_2_O of 99.9% atom D from ISOTEC (USA) was used. The real-time measurements started immediately after the thermal equilibrium was attained. Free induction decays (FIDs) were accumulated at 512–1024 times, corresponding to 1–2 h intervals. The digital frequency resolution was set to 0.3 Hz. Although D_2_O was selected as a solvent, the DANTE presaturation pulse sequence was applied to avoid the signal overlapping of impurity light water (HDO) with the target ^1^H NMR peak. The spectra were processed by the JEOL DELTA software. Chemical shift of the ^1^H NMR signal was obtained by referring to the absorption frequency of acetic acid in the solvent.

### Concentration analysis

Concentrations of reactant and product peptides were quantified by using integral intensities and/or peak heights of the respective NMR signals. For αA51–60, the signals of Asp58 H_α_ and Ser59 H_β_ was used. First, the amount of cleavage product, 51–58 fragment containing L-Asp was evaluated by using the integral intensities of the signal assigned to C-terminal L-Asp58 at 4.85 ppm and those of N-terminal Ser59 at 4.3 ppm, both relative to the initial concentration of the reactant. The loss of the reactant was evaluated by integral intensities of the non-terminal L-α-Asp58 signal at 5.1–5.0 ppm. Because the signal was partly overlapping with that of Phe53 at around 4.95 ppm, the concentration of non-terminal L-α-Asp58 was quantified by subtracting constant (time-independent) intensity of Phe53 H_α_ from the sum of the integrated signal intensities of non-terminal L-α-Asp58 and Phe53 from 5.08 to 4.91 ppm. Here, the integrated signal intensity corresponding to one proton of Phe53 was justified by using ring proton signal of Phe53 at 7.7–7.5 ppm corresponding to five protons. The intensities of non-terminal L-α-Asp58 were also estimated from the peak height at 5.02 ppm.

For non-terminal D-β-Asp58, the intensities were estimated from the peak height of H_α_ signal at 4.91 ppm because the signal was severely overlapping with C-terminal D-Asp58. Since the signal of C-terminal D-Asp58 at 4.85 ppm was weak and overlapped with non-terminal D-β-Asp58, the amount of C-terminal D-Asp58 was replaced by the concentration of N-terminal Ser59, obtained by integrating H_β_ peak at 4.33–4.27 ppm.

For αB61–67, the amount of the cleavage product was analyzed by using the integral intensity of methyl signal of N-terminal Thr63 in 63–67 fragment at 1.67–1.62 ppm. The assignment of Thr63 methyl signal was confirmed by the signal intensity that was three times as large as Asp H_α_, and by the doublet shape as a result of vicinal spin-spin coupling between methyl protons and the side chain proton. On the other hand, the loss of the reactant was evaluated by integral intensities of H_α_ of non-terminal L-α- and D-β-Asp62 at 5.14–5.06 and 4.99–4.91 ppm, respectively, as well as methyl signal of non-terminal Thr63 at 1.57–1.51 ppm.

### Population of staggered side-chain conformers

The population of staggered side-chain conformers (trans (T), gauche+ (G^+^) and gauche- (G^−^)) of αA51–60 and αB61–67 composed of L-α- and D-β-Asp residues was evaluated from vicinal spin-spin coupling constants H_α_-H_β1_ (*J*_αβ1_) and H_α_-H_β2_ (*J*_αβ2_) obtained by high-resolution ^1^H NMR measurement at 10 °C. Here, the temperature was as low as 10 °C to avoid undesired bond cleavage and intramolecular cyclization throughout the measurement. The detailed procedures are described elsewhere^21^. The digital resolution was as high as 0.02 Hz to obtain the coupling constants with high accuracy. FID signals were accumulated 2048 times.

Probabilities, *P* of each conformer, *P*(T), *P*(G^+^), and *P*(G^−^) were calculated from the following equations^25^,


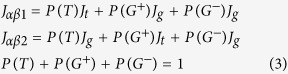
where *J*_t_ and *J*_g_ are the vicinal spin-spin coupling constants between α- and β-protons in pure T and G conformers. *J*_t_ (13.56 Hz) and *J*_g_ (2.60 Hz) were referred to Pachler’s parameter set^26^.

## Additional Information

**How to cite this article**: Aki, K. and Okamura, E. D-β-aspartyl residue exhibiting uncommon high resistance to spontaneous peptide bond cleavage. *Sci. Rep.* **6**, 21594; doi: 10.1038/srep21594 (2016).

## Supplementary Material

Supplementary Information

## Figures and Tables

**Figure 1:**
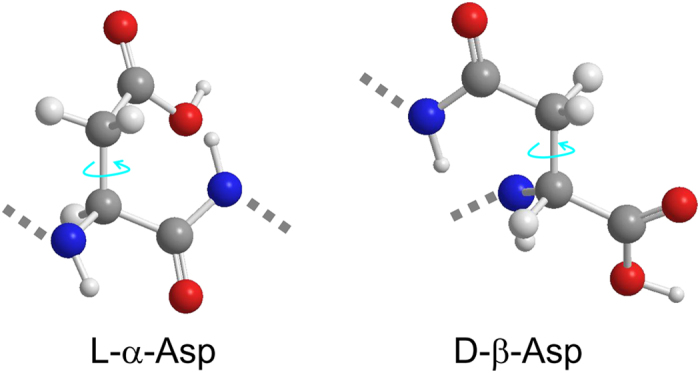
3D model of L-α- and D-β-Asp residues. Colors: C, gray; N, blue; O, red; and H, white. Notice that C_α_-C_β_ axes of L-α- and D-β-Asp side chains are rotated.
