# Supplementary material for: D-β-aspartyl residue exhibiting uncommon high resistance to spontaneous peptide bond cleavage
Source: Sci Rep. 2016 Feb 15;6:21594. doi: 10.1038/srep21594 (PMC4753488; doi:10.1038/srep21594)
Supplement: Supplementary Information [file srep21594-s1.pdf]

D-β-aspartyl residue exhibiting uncommon high resistance to spontaneous peptide bond cleavage

Kenzo Aki and Emiko Okamura

Supplement

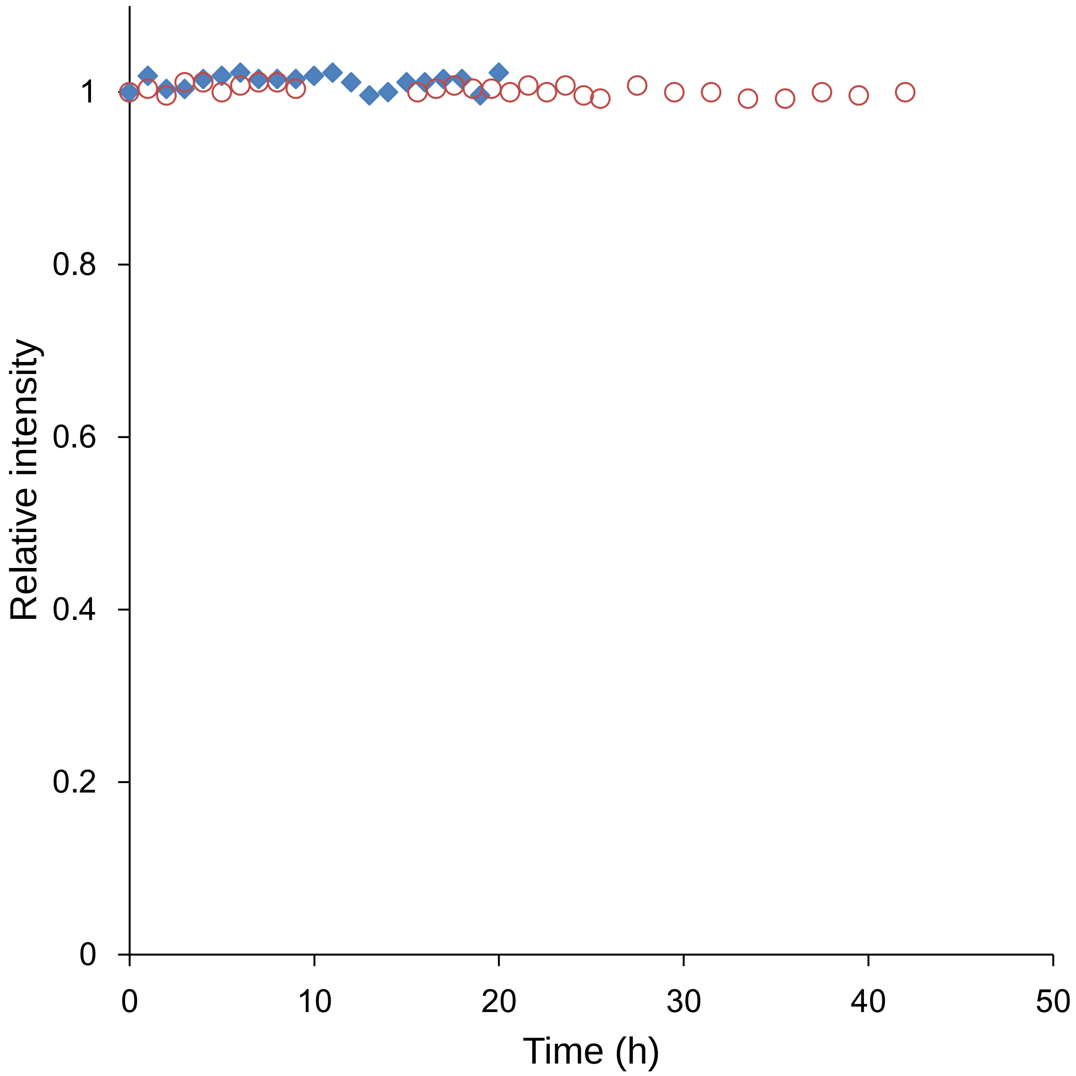

Figure S1. Real-time signal intensity change of Phe53 ring proton in αA-crystallin fragment, $S^{51}LFRTVLD^{58}SG^{60}$ during the bond cleavage reaction at L-α- (◆) and D-β-Asp58 (○). The intensities are the ones relative to the initial value at time 0.
